# Maternal Over- and Malnutrition and Increased Risk for Addictive and Eating Disorders in the Offspring

**DOI:** 10.3390/nu15051095

**Published:** 2023-02-22

**Authors:** Mathilde C. C. Guillaumin, Daria Peleg-Raibstein

**Affiliations:** Institute for Neuroscience, Department of Health Sciences and Technology, Swiss Federal Institute of Technology, ETH Zürich, 8603 Schwerzenbach, Switzerland

**Keywords:** maternal overnutrition, addiction, obesity, eating disorders, high-fat diet, offspring, animals

## Abstract

Evidence from human and animal studies has shown that maternal overnutrition and/or obesity are linked with neurobehavioral changes in the offspring. This fetal programming is characterized by adaptive responses to changes in the nutritional state during early life. In the past decade, an association has been made between overconsumption of highly-palatable food by the mother during fetal development and abnormal behaviors resembling addiction in the offspring. Maternal overnutrition can lead to alterations in the offspring’s brain reward circuitry leading to hyperresponsiveness of this circuit following exposure to calorie-dense foods later in life. Given the accumulating evidence indicating that the central nervous system plays a pivotal role in regulating food intake, energy balance, and the motivation to seek food, a dysfunction in the reward circuitry may contribute to the addiction-like behaviors observed in the offspring. However, the underlying mechanisms leading to these alterations in the reward circuitry during fetal development and their relevance to the increased risk for the offspring to later develop addictive-like behaviors is still unclear. Here, we review the most relevant scientific reports about the impact of food overconsumption during fetal development and its effect on addictive-like behaviors of the offspring in the context of eating disorders and obesity.

## 1. Introduction

Obesity, which is described by the World Health Organization (WHO) as a “global epidemic,” has become a preeminent public health problem due to multiple comorbidities including diabetes, cancer, and cardiovascular diseases [1,2,3]. In light of the overall increasing rates of obesity, it is not surprising that the prevalence of maternal overnutrition in the past three decades is on the rise [4]. It has been suggested that an individual’s susceptibility to obesity may originate early in life. This is the well-known “Barker Hypothesis”, which postulates that during early critical periods in development (i.e., during pregnancy), the organism has the ability to adapt to the environment and these prenatal adaptations are reflected in permanent changes in metabolic processes [5]. Epidemiological studies demonstrate a link between maternal overnutrition and increased predisposition to obesity, diabetes, metabolic disorders, and heart diseases in the adult offspring [6]. Leading to a deleterious cycle, these alterations in metabolic processes as a consequence of maternal overnutrition during this early developmental period are considered to have contributed to the epidemic rise in obesity. Although many argue that environmental factors (such as reduced physical activity, increased availability of highly-caloric food, increased size of food servings, etc.) play a significant role in developing obesity in adulthood, there is extensive evidence that environmental insults during pre- and postnatal development can predispose the offspring to become obese later in life [7]. However, it is incredibly complex to dissect in humans whether overconsumption of energy-dense foods high in added sugars and fat (the so called “junk food”) during pregnancy predisposes the child to obesity, or whether it is the eating habits learned in the household in early years which contribute to the child’s food preferences, ultimately resulting in obesity [8,9].

The overall goal of this review is to evaluate how far we have come in identifying, in animal models, the neural mechanisms underpinning the programming effects of maternal overnutrition on the reward system of the offspring leading to predisposition to addiction-like behaviors, obesity, and other eating disorders. Here, ‘maternal overnutrition’ includes high-fat and/or high-sugar, ‘cafeteria’ (consisting of a variety of highly-palatable, energy-dense foods such as cheese, chocolate, biscuits, ham, sausages, pâté, peanuts, crisps, golden syrup, cake, jam, etc.) and other calorie-dense diets. The maternal high fat diet (MHFD) rodent model is a powerful tool to dissect the complex priming effect of high-fat diets (HFD) and to identify mechanisms which render individuals susceptible to addiction and eating disorders. A MHFD does not necessarily imply a higher daily caloric intake, but a higher proportion of calories obtained from fat [10]. As the MHFD has been the most extensively used model in the literature (Table 1), it will receive special focus in the present review. A better understanding of the ‘re-programming’ mechanisms that can underlie these disorders has the potential to affect public awareness and preventative measures in humans with regard to these diseases, and to help identify new drug targets to combat both addiction to (or overconsumption of) palatable foods and drugs of abuse. Here, we will discuss findings on the effects induced by maternal overnutrition on the offspring’s reward system that might mediate the increased hedonic-like traits we and others have observed in response to palatable food and drugs of abuse, with an emphasis on the dopaminergic pathways (see Table 1 for references). This being said, we do not rule out that other neurotransmitter systems are involved or may even be the primary force underlying these neuropathologies. Indeed, given the wide array of behaviors among users in response to various drugs, it is likely that addiction is a multiple-neurotransmitter disorder in which dopamine only plays a partial role [11,12,13].

## 2. An Overview of the Role of the Dopamine System in Food Overconsumption

Food intake is largely governed by an intricate interplay between several neuronal circuits in the central nervous system [25]. Two of those, a homeostatic circuit in which the hypothalamus is a key area, and a reward-related circuit, play particularly crucial roles in regulating food consumption and energy balance [26,27,28]. The hypothalamus regulates homeostatic food intake and carefully matches caloric intake with energy expenditure to maintain stable body weight over time [29]. The natural reward circuitry, involving the dopaminergic circuit, which this review is focusing on, regulates the motivation to accomplish something desirable, such as the consumption of palatable food [30]. The reward system integrates dopaminergic neurons located in the ventral tegmental area (VTA), and the areas they project to, which include the nucleus accumbens (NAc) and regions of the prefrontal cortex (PFC); it also comprises dopamine neurons in the substantia nigra (SN), projecting to the dorsal striatum, which play a role in motivation [31,32]. These circuits regulate both the need to eat to survive and eating for pleasure. The dysregulation of either circuit can lead to overeating, accumulation of fat stores, and ultimately, obesity. Indeed, evidence from imaging studies have implicated limbic and cortical areas (e.g., NAc, PFC) as being involved in food overconsumption, beyond those directly regulating hunger and satiety [33,34]. Furthermore, these studies report that the release of dopamine (DA) in the striatum following ingestion of palatable foods is correlated with self-reported levels of pleasure derived from eating the food. Reduction in striatal DA signaling, such as decreases in the dopamine D2 receptors and DA release, have been linked to impairments in reward-processing in obesity [34,35]. Neuroimaging experiments conducted in obese and normal-weight individuals at risk for obesity show elevated reward circuitry responsivity in response to food cues as compared to lean subjects [34,35]. Strikingly, a similar picture emerges from brain imaging studies of drug-addicted humans [36,37]. Such parallels have generated significant interest in understanding the shared vulnerabilities and trajectories between drug addiction and food overconsumption [38,39]. An intriguing possibility is that dysfunctional DA circuits are the underlying cause for various types of addiction (alcoholism, compulsive gambling, overeating, and drug abuse). To date, there is no literature and no good experimental tools in humans that can demonstrate causal links between maternal overnutrition, neural circuit dysfunction (in particular DA circuitry), and substance addiction. Therefore, we need to rely on animal research. One might hypothesize that early life insults (such as maternal overnutrition) can alter the development of the dopaminergic circuits pre/postnatally, so that this will result in impaired DA signaling in adult offspring, leading to overconsumption of junk food and ultimately, obesity.

Animal models of maternal overnutrition have shown similar findings to those found in human studies in regard to increased risk of obesity [40,41], diabetes [41,42,43], cardiovascular disease (CVD) [44,45,46], increased emotionality [47,48], cognitive deficits [21,44,49,50,51], and neural alterations in the central nervous system [16,20,21,41,48,51,52,53] in offspring. These are arguments for animal models to be deemed adequate to study the impact of maternal diet on the offspring’s dopaminergic system and the risk of developing eating and addictive-like behaviors beyond metabolic effects. Although animal studies have advanced our understanding of how maternal overnutrition (induced by MHFD exposure) can lead to a broad spectrum of health adversities in the offspring, these mainly focused on metabolic traits. Thus, many questions remain open, in particular, the mechanisms via which maternal overnutrition increases the risk of developing addictive and/or eating disorders. We hypothesize that such mechanisms include early developmental alterations in the dopaminergic system in the offspring that become behaviorally evident later in life.

## 3. Peri-Gestation Maternal Overnutrition in Mice: A Robust Neurodevelopmental Animal Model of Several Behavioral Abnormalities

In the majority of animal studies examining the effects of maternal overnutrition, the mothers develop obesity prior to gestation and offspring are fed a HFD continuously throughout adulthood [54]. Thus, it is not always clear whether the effects in the offspring are a consequence of the maternal diet, a result from the current diet, or a combination of both. In contrast, an animal model has been developed where mothers are fed a HFD only pre-conception, during gestation and lactation, and do not develop obesity [20]. Offspring born to MHFD and control dams from this model are then fed normal chow from weaning onwards. We have reported that these MHFD offspring—exposed to normal laboratory chow from weaning through adulthood—develop an age-dependent metabolic phenotype, including increased body weight, cholesterol, triglycerides, and insulin levels, as well as increased fat composition compared to control offspring late into adulthood [20,50]. We have also shown that these nine weeks of HFD exposure (60% calories from fat) in female mice (beginning three weeks prior to mating and continuing through gestation and lactation) lead to a wide spectrum of behavioral abnormalities in the offspring such as reduced emotionality [48], cognitive impairments [21,50], and other neuropsychiatric disorders [20,21]. Several studies have found that those offspring also show increased preference in adulthood for foods high in added fats or sugars [14,16,20,21,53] and increased sensitivity to drugs of abuse [15,20,55].

**Figure 1:**
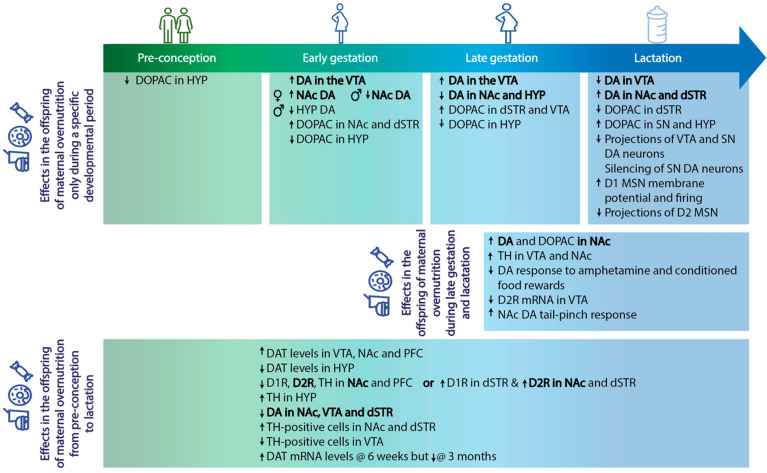
Animal models of maternal overnutrition (which includes high-fat, cafeteria—i.e., high-fat-high-sugar—diets, and which most closely mimic the obesogenic western diet in humans as a consequence of a tasty but unbalanced diet) during the periconceptional, gestational, and lactational periods have been key in our understanding of the non-genetic transfer of disease risk to the offspring. Exposure to these diets differs between studies, which results in dissimilarities (highlighted in bold) not only in maternal phenotype at the start of gestation, but also in changes in the central dopaminergic pathways in the offspring. These discrepancies of when maternal overnutrition exposure occurs can inform us about which periods of development are critical in inducing disorder susceptibility in the offspring and therefore, when to target interventions. D1/D1R: dopamine 1 receptor, D2/D2R: dopamine 2 receptor, DA: dopamine, DAT: dopamine transporter, DOPAC: 3,4-dihydroxyphenylacetic acid, dSTR: dorsal striatum, HYP: hypothalamus, MSN: medium spiny neuron, NAc: nucleus accumbens, PFC: prefrontal cortex, SN: substantia nigra, TH: tyrosine hydroxylase, VTA: ventral tegmental area, ↑: increase in, ↓: decrease in.

In addition, maternal overnutrition has been shown to lead to alterations in neural pathways critical in the regulation of behavior, particularly the dopaminergic system [20,21], in adult offspring. More specifically, alterations in DA transmission as well as DA markers have been reported in offspring exposed to MHFD [14,16,17,20,21] (Table 1 and Figure 1). In rodents, dopaminergic markers such as tyrosine hydroxylase, DA receptor density, DA content, and DA active sites gradually increase during the prenatal phase until the early postnatal phase (lactation) [For a review see [56]]. Thus, it can be speculated that dopamine neurons in the reward system are vulnerable to maternal overnutrition and this will alter behavioral and neural responses in the offspring, including overconsumption of palatable foods and addictive-like behaviors. The behavioral abnormalities in the adult offspring readily suggest that the perturbations caused by maternal overnutrition exposure are diverse and hinder processes fundamental to normal brain development and may involve other systems (beyond dopamine) such as the serotoninergic [48,57], GABAergic [48], and glutamatergic [48,50,51] systems, although these are not the focus of the present review.

In both humans and animals, it was shown that repeated access to highly-palatable food may eventually override the inhibitory processes that signal satiety, leading to compulsive consumption of large amounts of food despite nutrition overload [58]. As the mesolimbic DA system mediates the pleasure produced by natural rewards and drugs of abuse [59], it has been proposed that changes in DA transmission move an individual up and down along a “hedonic scale” [13]. The DA system is activated during consumption of palatable food. However, it is also shown to be activated in anticipation of food or rewards such as drugs [60,61,62]. This compulsive pattern of food intake is similar to that of drug intake patterns seen in addiction in two ways [63]: (i) by activating the reward system leading to over-eating and in turn, to prolongation of the meal and shifting the satiety sensation [30] and (ii) free access to palatable food also leads to adaptation [13] by shifting the homeostatic set points, resulting in obesity. In a series of studies, we have shown in the above-described MHFD mouse model that adult MHFD offspring prefer to consume sucrose and HFD more than control offspring [20,21]. One of the most significant findings was that when offspring were given free choice over several weeks to consume either a healthy diet (normal chow and water) or a palatable diet (HFD and sucrose solution) in their home cage, MHFD (but not control) offspring consumed significantly more palatable food and developed obesity and diabetes [20,21]. In addition, offspring born to MHFD-exposed dams were hypersensitive to the locomotor-augmenting effect of the dopamine indirect agonist, amphetamine, displayed increased alcohol intake, and showed greater conditioned place preference to cocaine [20,21]. These behavioral results have been hypothesized to be, at least in part, mediated by the same neuronal pathways and may be a consequence of an altered mesolimbic DA circuit, which is characterized by lower DA levels and enhanced striatal D1R and D2R expression [20].

Given the wide array of abnormalities that have been reported in the offspring from mothers fed a calorie-dense diet, this review will further focus on two groups of disorders: addiction-like behaviors and eating disorders, conditions that are overlapping, following maternal overnutrition, possibly via fetal reprogramming mechanisms.

## 4. Central Nervous System Alterations in An Overnutrition State Drive Addiction-Like Behaviors

Obesity is a condition characterized by metabolic abnormalities with many factors coming into play, among which are genetics, environment, exercise, diet, and other lifestyle practices [64]. Obesity was initially viewed as mainly mediated by impairments in peripheral signals, yet, it is now widely acknowledged that energy balance is achieved thanks to the interplay of a wide range of actors, among which the central nervous system (CNS) holds a key role [65]. Indeed, the CNS integrates various signals—hormonal and otherwise—about the body’s current energy state. Ultimately, once a pathological metabolic state has been reached, abnormalities within the CNS can further fuel a vicious cycle of addictive behaviors and food overconsumption [64,65,66].

Volkow and colleagues [31,61] hypothesized that common mechanisms underlie both drug and food addictions and that some forms of obesity, as well as substance addiction behaviors, may be mediated by imbalances in the dopamine system leading to changes in approach and reinforcer-sensitivity processing (although note that they do not exclude the involvement of other neurotransmitter pathways). Repeated consumption of foods high in added fat and sugar can lead to alterations in the dopaminergic pathways within the striatum (among which changes in D2 receptors regulation, with the direction of such changes still being debated), which hinders food-intake control exerted by the prefrontal cortex [31,33].

A study in mice also showed that recurrent exposure to sugar reduces the satiating effects of a caloric meal (directly administered in the stomach, in this study) [67]. This supports the idea that repeated exposure to sweet foods can drive overconsumption in a number of ways, among which a preservation of the appetite for sweet and caloric foods even when one’s stomach is full, suggesting either a misinterpretation of peripheral signals by the CNS or an altogether altered neurotransmitter and hormonal signaling in response to a meal. The common neurocircuitry underlying symptoms of drug addiction and food overconsumption in obesity or binge eating disorders (feeling of lack of control, absence of satiety, increased preoccupation for the addictive substance/food) supports the idea that obesity may be a form of ‘food addiction’ [31]. However, it remains controversial whether addictive-like feeding patterns can be considered a ‘true’ addiction [39,68,69]. A more crucial and long-standing question, as formulated by Montalvo-Martinez and colleagues [64], is: ‘are humans born addicted or do they become addicted to food?’. In the context of the current review, this means aiming to understand whether nutrition around and during gestation predisposes the child to food addiction or whether it is learned habits later in life that drive future food overconsumption. Evidence is already accumulating in favor of the former (although, of course, the latter is also a key contributor).

## 5. Maternal Overnutrition as A Risk Factor for Addiction

Direct associations between maternal overnutrition, substance abuse, and addictive behaviors have not yet been fully examined in humans, although there is increasing evidence for such associations in animal models [20,21,22,64]. Epidemiological studies have given evidence that maternal overnutrition can have long-term effects on the child’s behavior and can lead to abnormalities in the reward system in adolescents [24,52]. Animal studies on the other hand have shown that maternal overnutrition affects the DA neuronal circuits—although the direction of this impact is still unclear. Such effects may be mediated, among other factors, by elevated pro-inflammatory cytokines [70] and other inflammatory processes occurring in the mother and affecting the child’s neurodevelopment [64]. Unbalanced levels of metabolic hormones and nutrients driven by the nutritional state of the mother also affect the early development of the child’s brain, and particularly, the neural systems implicated in food intake and energy balance [71,72,73].

As mentioned earlier, there is a growing interest in studying ‘food addiction’ [74,75]; in light of the elements and evidence discussed above—showing the parallels between addiction to drugs and overeating—we consider this hypothesis plausible. Importantly, animal studies have shown that maternal high-fat-diet-induced obesity can be a risk factor for the offspring to develop substance or food addictions [52]. One could therefore draw another parallel here, given the mounting evidence that nicotine, alcohol, or cannabinoid consumption during pregnancy may prime the child to future addictions [76,77]. This further supports the idea that, whether overfeeding is considered a ‘true’ addiction or not, there are likely overlapping mechanisms with more conventional types of addictions.

Studies in rats have shown that maternal overnutrition during gestation can increase the likelihood for the offspring to binge on ethanol [78] or to become addicted to nicotine, as assessed by fixed-ratio and progressive-ratio paradigms, which are common assays to evaluate motivation to retrieve a reward [79]. Studies also support the idea that maternal overnutrition can lead to the development of tolerance to substances of abuse, such as amphetamine [15]. Beyond substance addiction, a few studies in both rodents and humans indicate a relationship between maternal overnutrition and child and adolescent food overconsumption and preference. In the laboratory, it was shown that infants from mothers with obesity tend to overeat foods with a high carbohydrate content [80]. Another study reported that mothers’ consumption of sweet food was related to earlier introduction of sweet foods and more frequent consumption of sweets by their 1-year-olds, with no differences in the consumption of high-fat or high-protein foods [81]. In rodents, studies have shown an increase in calorie-dense food intake in the offspring of mothers which had been exposed to such a calorie-dense diet during gestation and lactation, as compared to offspring from control mothers [53]. Other experiments corroborated this higher drive to consume highly-palatable foods, or of the rewarding properties of foods, in the offspring of over-nourished mothers [16,17,20].

However, disentangling the acute effects of maternal overconsumption during pregnancy with the effect of feeding habits after the child is born is complex. For example, a study suggested that maternal infant-feeding practices—i.e., after the child is born—impacts childhood obesity, but the mothers of the ‘high-risk group’ with higher obesogenic-promoting practices (e.g., introducing fruit juice and cereals to the bottle at infancy, 2 week- to 6-month-old babies) also had higher body mass index (BMI) themselves pre-pregnancy [82]. Thus, knowing to what extent the pre- and post-natal factors impacted the observed higher weight-to-length ratio in ‘high-risk’ infants is difficult. There is, generally, limited evidence indicating a possible relationship between maternal overnutrition and postnatal nutrition and child’s food preference and risk of overconsuming palatable foods.

Moreover, as discussed in a comprehensive review of the impact of maternal overnutrition on the risk for the child/offspring to develop neuropsychiatric disorders [52], obese mothers have higher systemic levels of various compounds (nutrients such as glucose, hormones such as leptin and insulin, as well as inflammatory markers) that can pass through the placenta and reach fetal circulation. Therefore, knowing whether the long-term effects in the offspring are due to a direct exposure to an unhealthy diet during early development, or to exposure to those imbalanced compounds due to a mother’s long-standing poor health (in the case of obesity pre-occurring to the pregnancy), has made it difficult to disentangle the mechanisms that underlie the neurophysiological effects of maternal overnutrition on the offspring. The authors of the above-mentioned review hypothesize that abnormal reward-related behaviors in the offspring of obese mothers is mediated by changes in the DA system, which would be triggered by inflammation.

## 6. Fetal Programming by Maternal Overnutrition and Its Impact on Addiction and Overeating in the Offspring

Another comprehensive review [64] has shown how maternal overnutrition can affect the health of the child, leading to a heightened risk of the child to develop metabolic conditions or psychiatric disorders via ‘fetal programming’. This mechanism includes various molecular and cellular alterations in response to a stimulus or insult during fetal development. Possibly allowing for adaptation initially (such as in Barker’s hypothesis regarding adaptation to fetal *under*nutrition [83]), it can lead to long-lasting changes in the physiology of the child, increasing the risk to develop chronic diseases, including metabolic conditions such as obesity and type II diabetes [84]. In rodent models, it was shown that maternal (over)nutrition programming can lead, for instance, to changes in the transcription of genes involved in glutamatergic pathways in the NAc and prefrontal cortex, leading to the offspring displaying an enhanced motivation to work for food and addiction-like behaviors in response to palatable foods [85].

Beyond transcriptional factors, an obese phenotype, like other states of inadequate nutrition, impairs immune function and leads to chronic inflammation via various mechanisms (e.g., increased production of pro-inflammatory leptin, endoplasmic reticulum stress due to nutrient excess, hypoxia-triggered expression of genes of inflammatory pathways, to name a few) [86]. It was shown in ewes that this inflammatory state may be passed on to the offspring, notably myocardial inflammation and fibrosis, alongside elevated plasma cortisol [87]. Elevated cortisol has been linked to acute stress response and acts to modulate inflammation, but chronic stress can lead to cortisol dysfunction and widespread inflammation [88]. As it does in the mother, inflammation triggered by maternal nutritional programming could in turn affect the offspring’s ability to sense metabolic signals both at the level of the brain and other organs (liver, pancreas, adipose tissue, etc.) [64,89]. It would thus impair metabolic homeostasis mechanisms, ultimately favoring the development of addictive behaviors around food in the offspring [64], but also sensitization to drugs [20,78]. It is interesting to note that drug addiction has been proposed to lead to inflammation and changes in the immune system which in turn affects DA neurotransmission [90,91]. This could be one more variable in how inflammation in pregnancy due to overnutrition can affect the reward system of the child and thus increase the risk to develop not only metabolic disorders but also addiction later in life. It was also suggested that changes in both satiety and inflammation pathways could fuel addictive behaviors, once again suggesting an interplay between the reward and homeostatic systems and an overlap between drug and food addictive behaviors [91].

As we have seen, maternal sugar consumption during pregnancy can impact the child’s metabolism, but also sweet taste perception and the increased risk of the child developing obesity later in life [92]. Intriguingly, both human and animal studies support the idea that paternal consumption of sugars can also affect the offspring metabolic processes, suggesting that epigenetic mechanisms come into play [92]—although this needs further investigation. However, indeed, epigenetic mechanisms are known to also mediate ‘fetal programming’. Infants from mothers with diabetes during pregnancy are at an increased risk to develop obesity, glucose intolerance, or type II diabetes themselves later in life [84,93]. Fetal ‘malprogramming’, via an altered placental metabolic milieu (e.g., placental glucose transport and fatty acid uptake, hyperglycemia, and elevated insulin), has been hypothesized to play a role in these increased risk factors in offspring [84,94,95]. Some molecular mechanisms are starting to be uncovered. For example, placenta analyses in mothers with type II diabetes (pre-existing to the pregnancy) or gestational diabetes have revealed epigenetic changes (methylation) in genes regulating mitochondrial function—a key player in metabolism, metabolic dysfunction [96], and inflammation [95,97].

In short, maternal overnutrition triggers adaptive responses in the offspring—affecting neuronal gene expression and immune/inflammatory responses, among others—that disrupt the brain reward pathways and set the stage for the development of a host of conditions, among which food addiction and compulsive eating disorders [64,91].

## 7. The Impact of Maternal Overnutrition on the Risk of Developing Eating Disorders

Studies in humans have provided some evidence for the role of maternal overnutrition in the development of eating disorders [52]. A recent review on the effect of eating disorders in mothers on the eating behavior of the child has shed light on the fact that children of mothers with an eating disorder are at higher risk to develop feeding and eating difficulties, alongside other psychopathological conditions [98]. A review in 2009 has shown that a maternal eating disorder during pregnancy could impact the child’s development—and the child’s future feeding and eating development—through various in utero mechanisms, including nutritional factors or increased glucocorticoids and other anxiety or depression-related hormones [99]. The authors argue that under- or overnutrition, as well as rapid variations in blood sugar levels, can negatively impact the development of the fetus, although the exact mechanisms are yet to be elucidated. Indeed, binging on high-glycemic index foods, possibly followed by purging which further disrupts normal hormonal responses to food intake, can trigger rapid oscillations in blood sugar and disrupt insulin responses; these oscillations in glycaemia and insulinemia (and other metabolic markers) can affect the child’s metabolism and development [99,100]. This is of particular concern in mothers with diabetes mellitus or gestational diabetes, of which the rates have been identified as particularly high in women with eating disorders or who are overweight or obese [101,102,103].

It was also shown that maternal overnutrition was correlated with inhibited and secretive eating habits in the child very early on (first 5 years) [104], which shows that those children are prone to developing concerns of weight gain at a very young age [52]. Other studies have shown that maternal bulimia nervosa (BN) and maternal binge eating disorder (BED) peri- and during pregnancy led to infants showing more disordered eating behavior [105,106]. Knowing that BN and BED have been shown to increase the intake of carbohydrate and sugar-rich foods during binges, such as bread, cakes, ice cream, candy, soft drinks, etc. [107], one cannot exclude that the *nutritional* effect of the mother’s eating disorder is affecting the offspring, beyond the post-natal effects whereby the child is impacted by the maternal (or paternal) *behavior* around food [108,109]. A study showed that mothers with BED before and during pregnancy had a higher total energy intake and a higher consumption of candy, fats, and milk desserts than controls [110]. Given that animal models of binge eating have shown that intermittent access to sugary, palatable foods trigger binge episodes [66,111,112], and that human literature also showed that binge episodes in humans are commonly triggered by the presence or consumption of highly-palatable food [111,113], one can wonder about the long-term effects of the repeated exposure in utero of the baby to those foods in pregnant mothers suffering from BN or BED. This is all the more concerning given that, as mentioned earlier, studies have shown that repeated exposure to sugars affects satiety-sensing mechanisms involving the dopamine dorso-striatal pathway [67]. Also of note, women with BN or BED during pregnancy had higher intakes of artificially sweetened beverages [110], possibly in an attempt to control caloric intake as part of their eating disorder. Although there is little direct clinical evidence regarding long-term harm caused by artificial sweeteners consumed during pregnancy on the child, a recent review has shown that consumption of artificial sweeteners before and during pregnancy led to increased infant weight and a heightened preference for sweet foods in the child [92]. In addition, there are controversial multi-system effects of artificial sweeteners on food intake and addiction (effects on the reward system, glucose homeostasis, or even microbiome health which is a key component of food metabolism) [114]. Cross-species animal experiments have shown that a mismatch between sweetness and energy intake when consuming artificial sweeteners (i.e., sucralose) can lead to increases in food intake via an increased motivation to eat, enhanced sweet taste perception, glucose intolerance, and a triggering of the ‘fasting response’ [115]. Bearing this in mind, the higher intake of artificial sweeteners in mothers with eating disorders during pregnancy is also a matter of concern for the child’s neurodevelopment.

**Figure 2:**
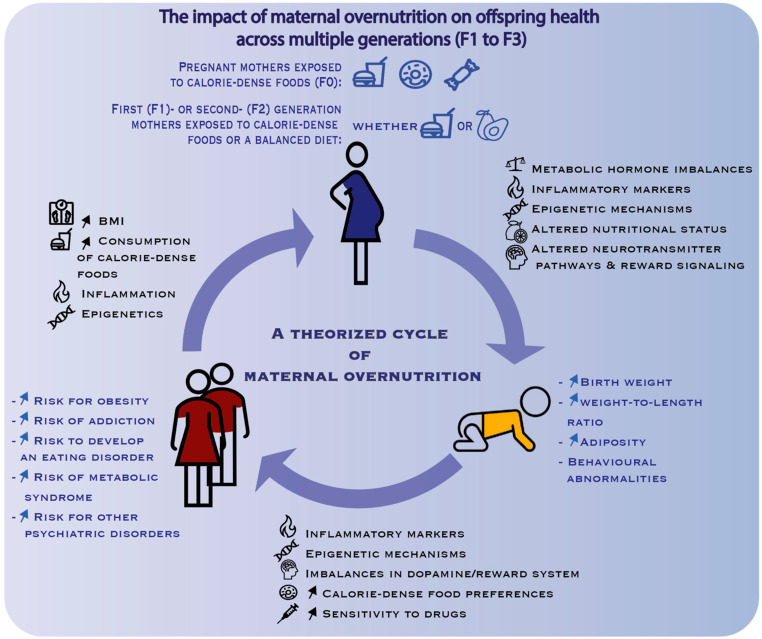
Summary of the possible mechanisms (in black) underpinning the negative effects (in blue) of maternal overnutrition on the offspring. The maternal overnutrition model is designed to test the impact of maternal (F0 generation) challenge with calorie dense foods on subsequent generations (F1, F2, F3) of male and female offspring consuming a standard chow. This schematic illustrates how such effects turn into a deleterious cycle as they are transmitted from one generation to the next. Note that peri-pregnancy overnutrition is necessary for the gestating female (F0 generation) but that the effects can be perpetuated in a transgenerational manner, even if the next-generation mothers (F1 which is the immediate offspring, second-generation (F2) offspring via the maternal or paternal lineage—i.e., male F1 offspring from over-nourished mothers are mated with naïve females to generate F2 offspring) eat a healthy diet during pregnancy. The epigenetic and other mechanisms leading to negative effects in the offspring can indeed also be transmitted by males (i.e., future fathers) to the next generation. F3, third generation offspring. ↑: increase in.

Finally, it was also shown that mothers with BN or BED before and during pregnancy had large-for-gestational age babies and babies with large birth length [103]. This is particularly concerning as other studies have shown that babies with large-for-gestational-age (LGA) lengths have a higher risk of developing obesity later in life and also have a higher risk of having large-for-gestational-age babies themselves, thus perpetuating a negative cycle of metabolic and/or eating disorders [116]. Importantly, the mothers’ own birth weight and adult BMI interact so that, for instance, a mother with a current healthy BMI sees her odds ratio of having an LGA infant climbing from 1 (if she had a normal birth weight) to 3.48 (if she was LGA herself); for a mother currently obese type II or III (BMI > 35), the odds ratio of her having an LGA child climbs from 3.86 (herself normal birth weight) to 14.14 (herself LGA) [116]. This further highlights the long-term effects of peri-gestational metabolic status on the health, not only of the child, but also on the descendants over several generations (Figure 2).

On a different side of the spectrum of eating disorders, anorexia nervosa (AN) has been associated with birth complications, lower birth weights in infants, and developmental alterations, all likely due to not only environmental and genetic factors, but also to nutritional factors, such as micronutrient deficiencies, as well as abnormal circulating hormone levels, including stress and appetite regulating hormones [109]. Additionally, it cannot be excluded that the self-induced starvation state of the mother may hold similar effects on child development and physiology to context-induced starvation as seen during the Dutch famine. Although there is little research to date looking at the metabolic status of children born from mothers with AN, studies of in utero undernutrition from the Dutch famine birth cohort have shown that starvation during pregnancy led to increased risk of the child developing metabolic and cardiovascular disease later in life [117,118,119]. Strikingly, it was also shown that children from this cohort had a higher likelihood of consuming a high-fat diet [120]. In women exposed to starvation in utero in the early stage of gestation, there was an increased risk of developing obesity [121]. In addition, effects of undernutrition prenatally had transgenerational effects. Children whose mothers had themselves been prenatally exposed to undernutrition had higher adiposity at birth [117,122] and children whose fathers had themselves been prenatally exposed to famine had increased BMI in adulthood [123]. It remains to be elucidated whether this deleterious cycle in the effects of prenatal under-eating may also hold true for the offspring of mothers suffering from AN during gestation. Just as for BN and BED, this would be important to elucidate in order to break the cycle of transgenerational disordered eating (Figure 2).

Properly disentangling genetic and environmental factors remains a challenge however, to understand what drives eating disorder onset in the children of mothers suffering from an eating disorder themselves [98]. To this day, there is very little research in both human and animals looking at the effect of maternal overnutrition on the risk for the child/offspring to develop eating disorders. Yet, the scarcity of such studies does not signify that there is no effect but should rather be viewed as an incentive to investigate these questions further. Given the ongoing obesity epidemic and the strong implications for the mental and physical health of future generations (without mentioning the societal and economic impacts it entails), we would gain from seeing such studies carried out as early as possible.

## 8. Conclusions

The impact of maternal over- and undernutrition during early development on the risk for the offspring to develop metabolic and behavior disorders is supported by an increasing amount of evidence. The fetal programming model suggests that a maternal nutritional imbalance, that can be either under- or overnutrition, has a long-term effect on the health of the offspring and on the risk of developing different health related disorders. In effect, not only maternal undernutrition, but also maternal overnutrition represents a form of malnutrition. However, particularly in the case of eating disorders, the underpinning mechanisms are largely yet to be discovered. It is crucial—given the prevalence of obesity and thus, of women becoming pregnant while being overweight, obese, or exposed to a hypercaloric diet—that we better understand the underlying mechanisms at a molecular and cellular level with a special emphasis on the central neuronal circuits being affected. This would allow us to devise adequate treatments and preventive and educational measures. If maternal overnutrition can drive the development later in life of metabolic diseases, substance addiction, eating disorders, and other neuropsychiatric disorders, to name a few, then surely, improving nutrition during pregnancy would be a well-invested effort. Not only would it help curb the obesity and metabolic disease epidemics, but it would also be valuable in helping against an increasing number of neuropathologies—from addiction to anxiety and depression [124]. Given the transgenerational effects of maternal overnutrition being increasingly unveiled (Figure 2), addressing these questions should be viewed as an urgency. Only then will we be able to break the pernicious cycle of metabolic disorders being passed on from one generation to the next and branching out to a host of other disorders.

## Figures and Tables

**Table 1:** Summary of findings in animals and humans relating to the effect of maternal overnutrition on the central dopaminergic system and addictive and eating behaviors. The diets used are high-fat (HFD) and cafeteria (mix of high-fat and high-sugar food products, which are commonly consumed by people) diets. In recent decades the maternal HFD has been most widely used in rodents. However, there are significant differences in the composition of the diets used where fat content (i.e., lard, plants, and fish) as well as the concentration (usually ranges from 30% to 60%) of the diets vary. In addition, the exposure to the diet also differs between studies which results in animal models with dissimilarities in maternal phenotype at the start of gestation. The age of the offspring when the measurements were performed also has an influence on the read-outs (see [14]). D1/D1R: dopamine 1 receptor, D2/D2R: dopamine 2 receptor, DA: dopamine, DAT: dopamine transporter, DOPAC: 3,4-dihydroxyphenylacetic acid, dSTR: dorsal striatum, HYP: hypothalamus, MSN: medium spiny neuron, NAc: nucleus accumbens, PFC: prefrontal cortex, SN: substantia nigra, TH: tyrosine hydroxylase, VTA: ventral tegmental area, ↑: increase in, ↓: decrease in.

Offspring Phenotypes					
Diet	Period	Changes in Central Dopaminergic Pathways	Changes in Addictive and Eating Behaviors	Species	References
**STUDIES IN RODENTS**					
HFD (30% fat)	Last week of gestation and whole of lactation.	↑ TH expression in the VTA & NAc. ↑ in DA and DOPAC (i.e., elevated DA tone) in the NAc. No significant changes in D1R, D2R or DAT levels.	↓ amphetamine-induced locomotion.	Rats	[15]
HFD (60% fat)	During pregnancy and lactation (introduction of HFD 60% 3 months before mating).	3-to-10-fold upregulation of the DA transporter (DAT) in the VTA, NAc and prefrontal cortex (PFC). Downregulation of DAT in the hypothalamus (HYP). Downregulation of D1R, D2R and TH in the NAc and PFC. Upregulation of TH in the HYP. DNA hypomethylation of the DAT gene promoter.	↑ preference for sucrose and fat.	Mice	[16]
HFD (30% fat)	Last week of gestation and whole of lactation.	Attenuated DA response to amphetamine in the NAc. ↑ activity of NAc synaptosomal DA transpoter sites. ↓ in VTA D2R mRNA levels. ↑ locomotor response to D2/3R activation.	↑ operant responding to obtain a fat-rich reward (but not a sugar-rich reward) in an FR/PR task.	Rats	[17]
‘Cafeteria junk-food diet’ (mix of sweet and fatty ingredients)	4 weeks pre-conception, gestation and lactation.	↓ NAc DAT mRNA levels during juvenile period (6 weeks of age). ↑ NAc DAT mRNA levels at adulthood (3 months of age). = VTA DAT mRNA levels. = TH, D1R or D2R mRNA levels in the NAc or VTA.	↑ fat and protein intake during the juvenile period. ↑ fat intake during adulthood.	Rats	[14]
HFD (30% fat)	Last week of gestation and whole of lactation.	↑ NAc DA response to acute tail-pinch. Alteration in DA response to both acute and repeated stress exposure.		Rats	[18]
HFD (30% fat)	Last week of gestation and whole of lactation.	↓ NAc DA response to food-reward-paired tone in a Pavlovian conditioning task. No difference in NAc DA levels between HFD and control offspring during food consumption in an unconditioned protocol.		Rats	[19]
HFD (60% fat)	3 weeks before conception, during gestation and during lactation.	↓ DA in the NAc, VTA and dorsal striatum. ↑ D2R levels in the NAc (core and shell) and dorsal striatum. ↑ D1R levels in the dorsal striatum. ↑ DAT levels in the medial prefrontal cortex. ↑ number of TH-positive neurons in the NAc (core and shell) and dorsal striatum. ↓ number of TH-positive neurons in the VTA.	↑ alcohol consumption. ↑ amphetamine-induced locomotion. ↑ cocaine-conditioned place preference. ↑ preference for sucrose and HFD.	Mice	[20]
HFD (60% fat)	3 weeks before conception, during gestation and during lactation.	↓ number of TH-positive cells in the VTA in F2 and F3 female offspring. ↓ levels of TH in the dorsal striatum (dSTR), NAc shell and VTA in F3 offspring. ↓ DAT expression in the NAc in F2 and F3 female offspring. ↓ D1R expression in the dSTR and mPFC in F2 female offspring. ↑ D1R expression in the dSTR and NAc in F3 offspring. ↑ D2R expression in the dSTR, NAc and mPFC of F2 offspring. ↑ D2R expression in the NAc (but decreased in the mPFC) of F3 offspring. ↓ DA levels in the VTA, NAc and dSTR in F2 and F3 offspring. ↓ levels of DOPAC in the NAc of F2 offspring and in the VTA of F3 female offspring.	3rd generation females show addictive-like behaviors. ↑ alcohol consumption, amphetamine-induced locomotion and cocaine-conditioned place preference in F2 and F3 generations (F3 males only showed ↑sensitivity to cocaine). F1 offspring showed ↑ sensitivity to both drugs of abuse and natural rewards.	Mice	[21]
HFD (60% fat)	Pre-conception (3 weeks prior to mating).	↓ DOPAC levels in the hypothalamus.		Mice	[22]
HFD (60% fat)	Early gestation (G0-G11).	↑ DA levels in the VTA. Females: ↑ NAc DA levels/ Males: ↓ NAc DA levels. ↓ DA levels in the Hypothalamus (males only). ↑ DOPAC levels in the NAc and dSTR. ↓ DOPAC levels in the hypothalamus.	↑ amphetamine-induced locomotion.	Mice	[22]
HFD (60% fat)	Late gestation (G12-G21).	↑ DA levels in the VTA. ↓ DA levels in the hypothalamus (females only) and NAc. ↑ DOPAC levels in the dSTR and VTA. ↓ DOPAC levels in the hypothalamus.	↑ alcohol preference. ↑ amphetamine-induced locomotion.	Mice	[22]
HFD (60% fat)	Lactation (3 weeks after birth, P0-P21).	↑ DA levels in the NAc and dSTR. ↓ DOPAC levels in the dSTR. ↑ DOPAC levels in the SN and hypothalamus.	↑ amphetamine-induced locomotion.	Mice	[22]
HFD (60% fat)	During lactation only.	↓ in projections of SN and VTA DA neurons. ↓ in DA release in the VTA. Silencing of SN DA neurons. ↑ in striatal D1 MSN membrane potential and firing. ↓ in projections of striatal D2 MSNs.	Females: ↑ in sucrose preference. Males: ↑ in novelty-induced locomotion.	Mice	[23]
**STUDY IN HUMANS**					
2 parents with BMI>27.	Not specified.	↑ activation of dorsal striatum in response to palatable food rewards. ↑ activation of striatum in response to monetary rewards.	↑ oral somatosensory response to palatable food rewards.	Humans	[24]
